# Human Immunodeficiency Virus Infection and Hodgkin's Lymphoma in South Africa: An Emerging Problem

**DOI:** 10.1155/2011/578163

**Published:** 2011-02-08

**Authors:** Moosa Patel, Vinitha Philip, Fatima Fazel

**Affiliations:** Division of Clinical Haematology, Department of Medicine, Chris Hani Baragwanath Hospital and the University of the Witwatersrand, P.O. Box 96092, Brixton, Johannesburg 2019, South Africa

## Abstract

Hodgkin's lymphoma (HL) occurs with increasing frequency in human-immunodeficiency-virus-(HIV-) infected individuals. The natural history and behaviour of HIV-HL is different, being more atypical and aggressive. The association between HIV and HL appears to be primarily EBV driven. HAART use does not significantly impact on the incidence of HL. Indeed, the risk of HL has increased in the post-HAART era. However, the advent of HAART has brought renewed hope, allowing standard therapeutic options to be used more optimally, with better treatment outcomes. Despite the renewed optimism, the overall survival of HIV-HL patients remains less favourable than that in HIV-seronegative patients. This is particularly true in sub-Saharan Africa, where there is a significant burden of HIV/AIDS and where more than half the patients are HAART naive at diagnosis of HL. The similarities and differences of a South African cohort of HIV-HL are presented in this paper.

## 1. Introduction

Human immunodeficiency virus (HIV) infection is known to be associated with an increased risk of Hodgkin's lymphoma (HL), based on linkage and cohort studies. The relative risk is now approximately 10-fold higher compared with the general population [1–5]. Earlier studies suggest that the HIV risk differed between the European and American cohorts with intravenous drug use being predominant in the Italian and Spanish patients compared to the American, where it was noted mainly in homosexual men [1, 2, 5, 6]. In sub-Saharan Africa, the major risk of acquiring HIV occurs with heterosexual relationships. This is true for all the patients in the series presented here (unpublished personal data; see Table 1).

HL occurring in the setting of immunodeficiency is generally aggressive, characterized by advanced-stage disease, frequent constitutional symptoms, less favourable histology, more frequent bone marrow involvement, and a poorer prognosis compared to immunocompetent individuals.

With the advent of HAART in 1996, the AIDS-related morbidity, particularly with respect to opportunistic infections, has decreased and the survival of HIV/AIDS patients has increased. HAART has allowed the use of standard therapeutic options to be delivered to seropositive patients in a more optimal manner, bringing about renewed optimism in the management of such patients. HAART use is associated with higher CD4 T-cell counts and enhanced immunity. Carbone et al. in 2009 [5] suggest that the improved CD4 T-cell count that occurs after HAART use provides antiapoptotic pathways and mechanisms for immune escape by tumour cells, thus resulting in an increased risk of HL [5].

A 20-year cohort study has shown that with the advent of antiretroviral therapy, ADCs (AIDS-defining cancers) continue to fall, but the rates of NADCs (non-AIDS defining cancers) are on the increase. The authors conclude that this increase appears to be more related to the aging of the HIV population rather than the antiretroviral therapy and its effect on the CD4 T-cell count [7]. 

This paper will focus on the epidemiology, pathogenesis, clinical presentation, and management of HL in the setting of HIV. The similarities and differences as observed in Southern African patients will also be highlighted.

## 2. Epidemiology

With the advent of antiretroviral therapy, ADCs (AIDS-defining cancers) continue to fall, but the rates of NADCs (non-AIDS defining cancers) such as HL, anal carcinoma, lung carcinoma, and skin cancers are on the increase [7]. With HL, there is a noticeably increasing relative risk of approximately 10-fold, compared with the general population [1–6]. 

The epidemiology of HIV-HL is comprehensively reviewed by Carbone et al. in 2009 [5]. In this excellent review, the authors summarise the findings of several epidemiological studies conducted in the last two decades, which strongly support the evidence that HIV-positive individuals have a higher risk of developing HL compared to their HIV-negative counterparts. This is in contrast to HIV-NHL or HIV-Kaposi's sarcoma (where the incidence of the disease has decreased significantly after the introduction of HAART). Thus, despite the benefit of HAART, which improves immunity and decreases the risk of opportunistic infections, there is a paradoxically increased risk of HL [5].

More than two thirds of all people with HIV live in sub-Saharan Africa, and South Africa is home to the world's largest population of people living with HIV—5.7 million in 2007 [8]. Early studies in the mid 1990's and early 2000's in South Africa (including patients from the Chris Hani Baragwanath Hospital (CHBH)—a tertiary, public sector hospital, linked to the University of the Witwatersrand, and located in Soweto, Johannesburg) showed only a modest increase of Non-Hodgkin's Lymphoma (NHL) in seropositive individuals, with odds ratios of 4.8, 5, and 5.9, respectively [9–11].

However, at CHBH, since 2002, there has been a clearly noticeable increase in the proportion of seropositive patients with NHL (i.e., 60–70% of newly diagnosed patients with NHL are HIV positive), and furthermore, there has been a significant increase in the total number of patients with NHL since 2002 (from 20–30 new patients per year to 70–80 patients per year) [12]. Indeed, NHL is now the commonest haematological malignancy in South Africa, in the current HIV/AIDS era.

With respect to Hodgkin's lymphoma (HL), the data is less dramatic but is becoming more significant. In a study by Stein et al. in 2008 [11], the percentage of seropositivity of HL in a South African cohort (which included patients from CHBH) was 19.5% (OR = 1.6, 95% CI = 1.0–2.7), during the period from 1995 to 2004. However, in the last 4 years, at our single institution, CHBH, the percentage of seropositivity has been greater than 50% (unpublished personal data) and the number of patients over the years has been gradually increasing (doubled compared to an earlier series in the late 1980's and early 1990's) [13]. In the last 2 years (July 2008 to June 2010), a total of 43 consecutive adult black patients with HL were diagnosed at this single institution. Of these patients, 29 were HIV seropositive (67%) and 14 (33%) were seronegative. The clinical characteristics of this retrospective review of the seropositive patients are depicted in Table 1.

## 3. Pathogenesis

HL is characterized by an admixture of a minority of neoplastic Reed-Sternberg (RS) cells and a reactive, mixed inflammatory infiltrate of lymphocytes, plasma cells, eosinophils, and histiocytes. Cytokines and chemokines are produced by either the RS cells or the reactive cells. The cytokine production may explain the presence and maintenance of an impaired immune response, while the chemokines (cytokines with chemoattractant properties) play a role in leucocyte trafficking, attract chemokine receptor CCR4-expressing Th-2 cells and T regulatory cells, and allow a favourable environment for survival of RS cells [14–16]. Cross-talk between the RS cells and reactive cells mediated by cytokines such as IL-13, IL-17, IL-10, transforming growth factor beta, and chemokines,principally CCL17 (thymus-and activation-regulated chemokine, TARC) and CCL22 (macrophage-derived chemokine, MDC), leads to an environment where RS cells are able to proliferate, escape from apoptosis, and survive host antitumour defense [14–16]. The CD4+ T cells surrounding the neoplastic cells in HL are CD45RO+/CD45RA-/CD45RB^dim^, suggesting a memory Th2 phenotype [17].

HIV-associated immunosuppression is a state that permits the unchecked and uncontrolled proliferation of Epstein-Barr virus (EBV) infection. EBV has been implicated in the aetiopathogenesis of classic HL. EBV-transforming proteins, such as latent membrane protein-1 (LMP-1), is expressed in virtually all HIV-HL patients [18–20]. The expression of EBV-LMP-1 is important in the pathogenesis of HIV-HL. LMP-1 expression by EBV-infected RS cells represents the principal mechanism for constitutive nuclear factor NF*κ*B activity, which confers an apoptosis-resistant phenotype to the RS cells [19, 20]. EBV-immortalized B cells also produce CCL17 and CCL22 through LMP-1-mediated activation of NF*κ*B [21]. 

RS cells represent transformed B cells (postgerminal center B cells) that originate from preapoptotic germinal center B cells. They express LMP-1 and display a BCL6-/CD138+/MUM1/IRF4+ (Interferon Regulatory Factor-4) phenotype [19, 22, 23]. In addition, LMP2A and EBNA-1 may also contribute to the development of the RS cells, and they are expressed in the RS cells of this tumour [18, 20]. LMP2A may promote the survival of the “crippled” germinal center B cells, thereby aiding in their development [24].

## 4. Clinical Presentation and Management

HIV-seropositive patients with HL generally pursue a more aggressive clinical course than seronegative patients. The behaviour of the disease is different, and based on a number of studies [5, 6, 25–32], the following parameters were noted: more advanced-stage disease (III and IV), 74–92%, more frequent systemic B symptoms, 70–96%, more frequent involvement of extranodal sites, 17–62%, with bone marrow involvement being the most common extranodal site, 40–59%, followed by involvement of the liver, 17–40%, and spleen, 20–30%. Most of the patients (>80%) were males. The median age at presentation was approximately 34 years. The median CD4 count was mostly in the intermediate range of 240–306/*μ*L [5, 6, 25–32]. Compared to HIV-negative HL, where nodular sclerosis is the dominant histological subtype, mixed cellularity is most commonly encountered in HIV-HL, 33–53% [5, 13, 30–32]. Nodular sclerosis is the second most common histological subtype in HIV-HL, 24–31%. However, with more severe immunosuppression, nodular sclerosis becomes infrequent [33]. There is also an increasing number of patients with lymphocyte-depleted histology, 14–20%, in HIV-HL [5, 30–32].

Based on the Italian Cooperative Group on AIDS and Tumors (GICAT), in comparison with patients who were HAART naive, patients receiving HAART before the onset of HL are older, have less B symptoms, have higher leukocyte and neutrophil counts, and have a higher haemoglobin level [30].

The characteristics of the patients with HIV-associated HL seen at CHBH over a 2-year period are depicted in Table 1. There are some striking similarities and differences when comparing this cohort with other published studies outside of Africa. The median age at presentation is similar. There is no striking male predominance in our patients. Conversely, the male to female ratio is almost equal at 1.1 : 1. None of our patients have homosexual contacts or are intravenous drug users. This is different from other series where homosexuality and intravenous drug use are significant, documented risk groups [1, 2, 5, 6, 30, 31]. The presentation with advanced-stage disease, more frequent systemic B symptoms, more frequent involvement of extranodal sites, and the histological patterns of disease is similar to that reported in the literature (see above). The median CD4 count of 176/*μ*L is generally lower in our patients, although there are series reported of HIV-HL with median CD4 counts of <200/*μ*L [31]. In our series, 12/29 (41%) of the patients had newly diagnosed HIV at the time of the diagnosis of HL. In 62% of the patients, the duration of the diagnosis of HIV (including new patients) was <1 year. Only 45% of the patients were on antiretroviral therapy at diagnosis of HL, compared to 71–80% in other series [5, 30, 31]. A further striking difference is the high proportion of patients with Tuberculosis in our series,59% (38% with active disease and 21% with past, documented disease). The high prevalence of tuberculosis may be a reflection of the more severe immunosuppression in our patients, the delay in diagnosis of HIV, and hence the absence of antiretroviral therapy use at diagnosis, and the common occurrence of tuberculosis in the general population. The presence of tuberculosis, often in a disseminated fashion, impacts adversely on the clinical outcome of our patients.

The salient differences between HIV-HL in South Africa and other reported series outside of Africa (mostly from Europe and the USA) [5, 6, 25–32] are detailed in Table 2 above.

The management of HIV-HL is challenging because of the frequency of infections, likelihood of organ dysfunction due to HIV, more frequent involvement of the bone marrow, increased myelosuppression, potential interactions of the antiretrovirals and anti-infectives with chemotherapy, the advanced and widespread nature of the disease at presentation, and the preponderance of less favourable histological subtypes. Treatment approaches include vigorous supportive care (HAART, antivirals, antifungals, neutrophil-stimulating growth factors), together with standard multiagent chemotherapy. 

Chemotherapy regimens for HIV-HL such as ABVD, EBVP, BEACOPP, MOPP/ABV hybrid, and Stanford V are feasible and can be delivered with concomitant HAART. The AIDS Clinical Trials Group (ACTG) treated 21 patients with ABVD for 4–6 cycles and primary use of G-CSF. Antiretroviral therapy was not used. The complete remission rate, on intent-to-treat analysis was 43% with an overall objective response rate of 62%. Median survival for all patients was 18 months [34]. In a more recent Spanish study, 62 patients with HIV-HL received the standard, full-dose ABVD and HAART. 87% achieved a complete remission. The 5-year overall survival (OS) and event-free survival (EFS) probabilities were 76 and 71%, respectively. The immunological response to HAART had a positive impact on OS (*P* = .002) and EFS (*P* = .001) [35]. The use of HAART substantially improves the overall survival in HIV-associated HL. This is due to a decrease in the incidence of opportunistic infections, the ability to deliver more appropriate and aggressive chemotherapy on schedule, and to the less aggressive presentation of lymphoma in patients on HAART, in comparison with those lymphomas that arise in patients who never received HAART [6, 25–27, 29]. In the study by Hentrich et al., in 2006, 34/59 patients receiving HAART (*n* = 34) had a significantly better 2-year overall survival than those not receiving HAART (74% versus 30%, *P* < .001) [36]. The advent of HAART also allows for more aggressive treatment options such as VEBEP [37], BEACOPP [38], and Stanford V [39] and the use of high-dose chemotherapy and autologous stem cell transplantation (ASCT) in selected patients [40, 41]. However, in general, response rates and cure rates are lower than in HIV-seronegative patients, despite the substantial progress made in the last decade. The challenge at present is to optimise the use of standard approaches as used in HIV-negative HL. Once this is established, evaluation of experimental and newer therapies should follow.

## 5. Conclusion

HIV is associated with an increased risk of HL. Although the risk is much less than with NHL, the risk appears to be increasing with time, with HL now being regarded among the most common NADCs. The increased incidence cannot be explained by more intensive surveillance or diagnostic errors [42]. The association, which was being largely coincidental (overlapping and similar age group for both HL and HIV) may now be increasingly causal, with the most plausible explanation being attributed to the pathogenetic role of Epstein-Barr virus infection. Therapy of HIV-associated HL entails using the same therapeutic approaches as in seronegative HL, including standard chemotherapy regimens such as ABVD, and in the salvage setting, autologous stem cell transplantation in selected patients. In general, the prognosis and overall survival are poorer in HIV-HL compared to HIV-negative HL. Importantly, the concomitant use of antiretroviral agents and prophylaxis against certain opportunistic infections such as Pneumocystis jiroveci pneumonia, as well as the liberal use of growth factors (granulocyte colony stimulating factor) and other supportive measures, constitutes an important aspect of supportive therapy and has contributed to an improvement in prognosis. The early recognition and treatment of tuberculosis cannot be overemphasized in settings where tuberculosis is endemic. Newer specific treatment approaches for HL may become necessary to improve survival, as the association with HIV increases and becomes clearer in the future. For the present, HIV-associated HL remains an ongoing challenge.

## Figures and Tables

**Table 1:** Characteristics of patients (*n* = 29) with HIV-associated Hodgkin's lymphoma seen at the Chris Hani Baragwanath Hospital (July 2008 to June 2010).

Patient characteristics	Number	%
Median age (range)—38 years (21–64) mean age—37 years (21–64)		
Gender		
Male	15	52
Female	14	48
Male : female ratio 1.1 : 1		
ECOG performance status		
0–2	18	62
3-4	11	38
“B” symptoms	27	93
Bone marrow involvement		
Yes	10 (26)	38
No	16 (26)	62
Unknown	3	11
Stage of lymphoma		
I-II	5 (28)	18
III-IV	23 (28)	82
Unclear	1	4
Histology		
Mixed cellularity	13 (24)	54
Nodular sclerosis	8 (24)	33
Lymphocyte depleted	2 (24)	8
Lymphocyte rich	1 (24)	4
Unclassifiable/unknown	5	17
CD4 count in *μ*l (range in *μ*l)—mean CD4 = 176 (10–407)		
>200/*μ*l	11 (28)	39
<200/*μ*l	17 (28)	61
<100/*μ*l	9	31
<50/*μ*l	6	21
Unknown	1	3
Risk factor for hiv infection		
Heterosexual relation	29	100
Other (intravenous drug use, homosexual relation)	0	
Duration of hiv		
>1 Year	11	38
<1 Year	18	62
Use of antiretroviral therapy at diagnosis		
Yes	13	45
No	16	55
True extranodal involvement (excluding liver, spleen, and bone marrow)	5	17
Liver involvement	13	45
Splenic involvement	8	28
Tuberculosis	17	59
Active disease	11	38
Past infection	6	21
Outcome		
Alive	15	52
Dead	11	38
Ltfu (lost to follow up)	3	10

**Table 2:** Salient differences between HIV-HL in South Africa and other reported series.

	South Africa (Chris Hani Baragwanath Hospital—CHBH)	Other reported series
Gender	M : F ratio,1.1 : 1	Majority male (>80%)
Median CD4 count	176/*μ*l	240–306/*μ*l (In different series)
Risk factors	Heterosexual relationship	Intravenous drug use and homosexual relationship
Diagnosis and duration of HIV	New diagnosis of HIV, 41%	Majority-established or long-standing HIV
	Duration of HIV <1 Year, 62%	
ARV's at diagnosis	45% on ARV'S	71–80% on ARV'S
	55% ARV naive	
Tuberculosis	38% Active disease, 21% past, documented disease	Unknown (low prevalence)
Prognosis and overall survival	Generally unfavourable	Less favourable than HIV negative HL. Improving with standard chemotherapy and concomitant HAART
